# Cellular enlargement - A new hallmark of aging?

**DOI:** 10.3389/fcell.2022.1036602

**Published:** 2022-11-10

**Authors:** Daniel M. Davies, Kim van den Handel, Soham Bharadwaj, Jette Lengefeld

**Affiliations:** ^1^ Institute of Biotechnology, HiLIFE, University of Helsinki, Helsinki, Finland; ^2^ Center for Hematology and Regenerative Medicine, Department of Medicine Huddinge, Karolinska Institutet, Stockholm, Sweden

**Keywords:** Cellular enlargement, hallmarks of aging, cell size physiology, aging-related diseases, stem cells

## Abstract

Years of important research has revealed that cells heavily invest in regulating their size. Nevertheless, it has remained unclear why accurate size control is so important. Our recent study using hematopoietic stem cells (HSCs) *in vivo* indicates that cellular enlargement is causally associated with aging. Here, we present an overview of these findings and their implications. Furthermore, we performed a broad literature analysis to evaluate the potential of cellular enlargement as a new aging hallmark and to examine its connection to previously described aging hallmarks. Finally, we highlight interesting work presenting a correlation between cell size and age-related diseases. Taken together, we found mounting evidence linking cellular enlargement to aging and age-related diseases. Therefore, we encourage researchers from seemingly unrelated areas to take a fresh look at their data from the perspective of cell size.

## 1 Introduction

Aging is the time-dependent loss of physiological integrity (Lopez-Otin et al., 2013) that can lead to numerous diseases like neurodegenerative diseases, cardiovascular disease, autoimmune diseases and cancer (Sadighi Akha, 2018; Stauder et al., 2018; Hou Y. et al., 2019; Rodgers et al., 2019; Zjablovskaja and Florian, 2019). The appearance of some of these diseases is facilitated by aging-related stem cell dysfunction. One of the most significant examples of this are HSCs. HSCs possess the ability to self-renew and are multipotent, thereby giving rise to the multiple different cell types of the blood. A healthy blood system requires HSCs to balance and maintain both these abilities, which decline with age. This leads to stem cell exhaustion and biased lineage differentiation (Yamashita and Iwama, 2022), which can contribute to the development of anemia, leukemia and a compromised immune system (Dykstra and de Haan, 2008; Hill et al., 2017; Chen et al., 2019; Chopra and Bohlander, 2019; Anurogo et al., 2021).

Our current understanding of aging splits into nine categories known as the hallmarks of aging (Lopez-Otin et al., 2013): cellular senescence, mitochondrial dysfunction, stem cell exhaustion, telomere attrition, altered intercellular communication, deregulated nutrient sensing, loss of proteostasis, chromatin remodeling, and genomic instability. Each category represents a factor that 1) manifests during aging, 2) upon accumulation accelerates aging 3) and upon removal slows down aging (Lopez-Otin et al., 2013). By now these hallmarks are almost a decade old, and it is unclear whether additional hallmarks of aging exist.

Since the 1960s, it has been known that cells become large *in vitro* when entering the senescent state (Hayflick and Moorhead, 1961). Several researchers also observed that cells *in vitro* and *in vivo* enlarge during aging (Hayflick and Moorhead, 1961; Mitsui and Schneider, 1976a; Mitsui and Schneider, 1976b; Treton and Courtois, 1981; Martinelli et al., 2006a; Mammoto et al., 2019). Since then, tremendous efforts have been made towards understanding how cells regulate their size. However, it was not until recently that we gained a deeper understanding of why cells regulate their size (Miettinen and Bjorklund, 2016; Neurohr et al., 2019; Lengefeld et al., 2021). Focusing on HSCs, we discuss the implications of these recent findings and the potential of cellular enlargement as a new hallmark of aging (Lengefeld et al., 2021). Then, we highlight numerous publications reporting a correlation between cell size and diseases of old age. We expose the need for future research to address whether these correlations are reflecting a causal relationship between enlargement and function, and the implication this has on research areas that have so far not considered the importance of cell size.

## 2 Cellular enlargement: A new hallmark of aging?

A large body of literature highlights two important findings: 1) Different cell types display different average sizes (Ginzberg et al., 2015) and 2) cells maintain a uniform size by using several regulatory pathways (Lloyd, 2013; Cadart et al., 2014; Schmoller et al., 2015; Amodeo and Skotheim, 2016; Schmoller, 2017; Bjorklund, 2019). This raises the question of why cells invest in maintaining their size. Therefore, understanding what happens when cells fail to regulate their size is important. While the first findings around this topic led to controversial conclusions (Bilinski, 2012; Ganley et al., 2012; Kaeberlein, 2012), budding yeast has been a key model organism to provide the first evidence that cellular enlargement could be directly linked to cellular dysfunction during aging (Bilinski and Bartosz, 2006; Zadrag-Tecza et al., 2009; Yang et al., 2011; Bilinski et al., 2012; Neurohr et al., 2019). It is known that budding yeast cells enlarge during aging (Mortimer and Johnston, 1959; Yang et al., 2011; Bilinski, 2012; Lee et al., 2012; Denoth Lippuner et al., 2014). Preventing this enlargement with drugs preserves their replicative age (Johnson et al., 2013; Neurohr et al., 2019). Similarly, preventing cellular enlargement *in vitro* in primary human cells has been shown to maintain their capacity to enter the cell cycle thereby avoiding cellular senescence (Demidenko and Blagosklonny, 2008; Demidenko et al., 2009; Neurohr et al., 2019; Lanz et al., 2022).

Our recent publication dissected whether the role of cell size on cell function is based on correlation or causation. An intrinsic challenge was to manipulate cell size without targeting other pathways, and to delineate that the observed changes are causal and not correlative. This hurdle was tackled using HSCs *in vivo* (Lengefeld et al., 2021; Strzyz, 2022):

• Six orthogonal approaches were examined under which HSCs became larger. In each of these conditions, HSC function was also compromised. HSC function was determined as their ability to form a blood system after transplantation into recipient mice. While it could be argued that each single manipulation affected HSC function unrelated to cell size, together these experiments suggest that the dysfunction was not driven by an unaccounted variable. Furthermore, alternate causes were excluded by analyzing other parameters of the hematopoietic system: homing, stem cell identity, differentiation potential and cell cycle state. Therefore, the simplest explanation is that enlargement of HSCs reduces their functionality.
• Treatment with a Cdk4/6 inhibitor (palbociclib-PD) caused artificial HSC enlargement and dysfunction. In PD-treated animals, not all HSCs became larger and those HSCs that stayed small displayed higher functionality than large HSCs from PD- or vehicle-treated mice. This indicates that PD-induced enlargement, rather than PD-treatment *per se,* causes HSC dysfunction.

Further lines of experimental evidence from this study support the conclusion that cellular enlargement causally decreases HSC function (Lengefeld et al., 2021):

• Preventing HSC enlargement by inhibiting macromolecule biosynthesis (rapamycin treatment) during insults that enlarge HSCs (DNA damage, successive divisions and aging), protected HSCs from losing their stem cell function.
• Reducing the large size of non-functional HSCs by shortening G_1_ (*RB* mutation) restored their functionality. Removal of *RB* does not improve the function of control or small HSCs (Walkley and Orkin, 2006; Lengefeld et al., 2021), indicating that the lack *RB* does not improve stem cell function *per se*, but restores it specifically by reducing cell size.

Together these data make a strong case that enlargement drives the dysfunction of murine HSCs *in vivo*.

**FIGURE 1:**
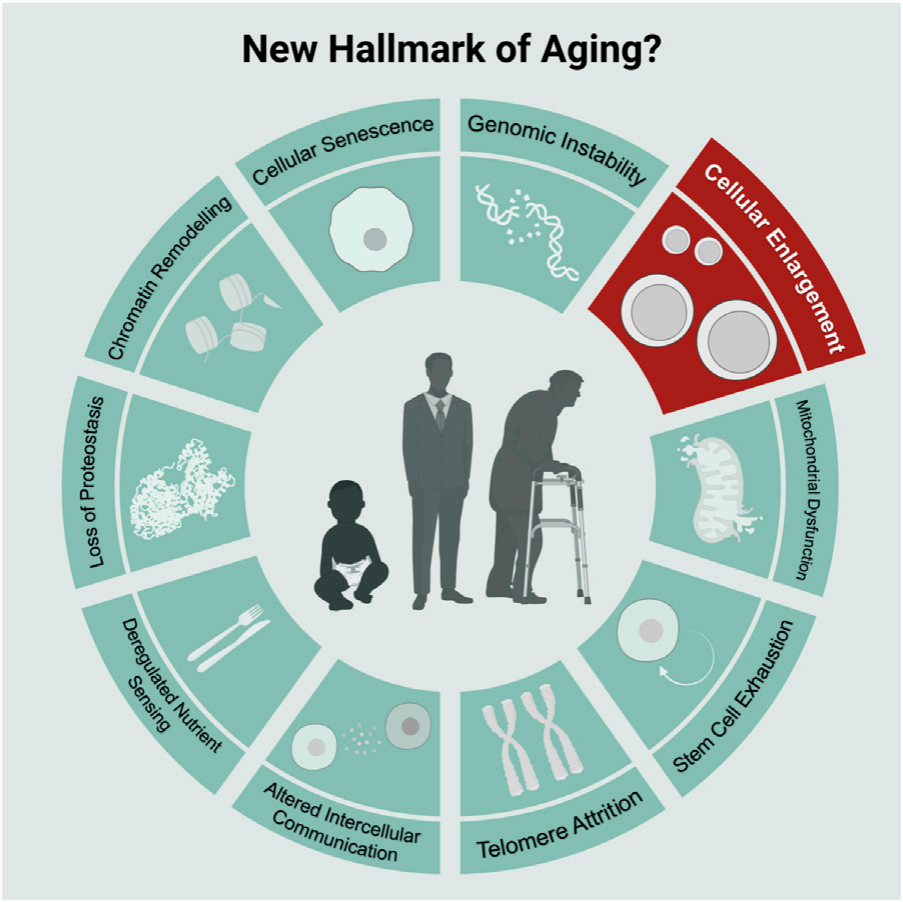
Scheme of commonly known nine hallmarks of aging in light-green and the new hallmark “Cellular Enlargement.” Scheme adopted from Lopez-Otin et al. (2013) and created with BioRender.com.

In addition to the above-listed observations, cellular enlargement also qualifies as a novel aging factor for HSCs (Figure 1): 1) HSCs naturally enlarge during aging, 2) artificially increasing cell size reduces HSC function and induces the appearance of aging characteristics and 3) preventing HSC enlargement during aging using rapamycin preserves the function of HSCs (Lengefeld et al., 2021). To thoroughly evaluate whether cell enlargement qualifies as an *bona fide* aging factor/hallmark, it will be important generalize these studies by testing for a causal relationship between enlargement and dysfunction also in other stem cells and even differentiated cells.

Of note, accumulating evidence suggest that small size is important for stem cells in general. Like HSCs, mesenchymal stem cells enlarge during aging *in vitro* (Oja et al., 2018), intestinal stem cells display size dependent functions (Lengefeld et al., 2021) and numerous stem cell types are small in size (Colter et al., 2000; Colter et al., 2001; Smith et al., 2004; Young et al., 2004; Virant-Klun et al., 2008; Zuba-Surma et al., 2008; Virant-Klun and Stimpfel, 2016). Thus, it is likely that mechanisms connecting cellular size with aging-related function are conserved in other stem cell types as well.

**FIGURE 2:**
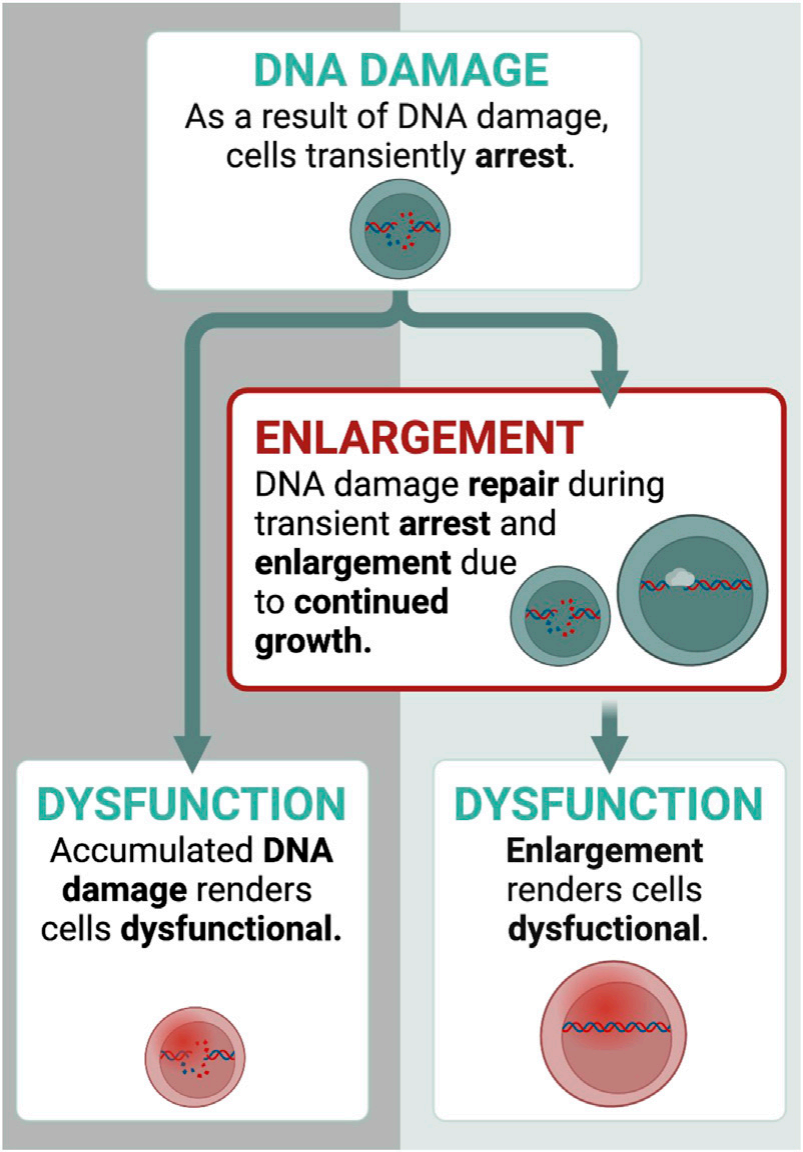
How do HSCs enlarge? Transient cell cycle arrests drive enlargement of HSCs. Here, we display an example with DNA damage. DNA damage affects HSCs in two ways: 1) it causes dysfunction directly 2) and transiently arrest HSCs for DNA damage repair, during which growth continues. This leads to the enlargement of HSCs. Once they are too large, they become dysfunctional. Thus, DNA damage contributes to dysfunction also indirectly by enlarging HSCs. Created with BioRender.com.

How do HSCs enlarge during aging? To explain the process of HSC enlargement and the consequent loss of their function during aging we proposed the following model (Lengefeld et al., 2021): As HSCs divide and age, they experience conditions that cause cell cycle arrest. For example, DNA damage incurred during replication (Geiger et al., 2013; Flach et al., 2014; Walter et al., 2015) activates cell cycle checkpoints that transiently halt division until the DNA damage is repaired (Sperka et al., 2012; Moehrle et al., 2015). During arrest phases, cell growth continues leading to increased HSC size (Fingar et al., 2002; Neurohr et al., 2019; Padovani et al., 2022). The enlarged HSCs become dysfunctional. Indeed, inhibition with rapamycin prevents enlargement during these arrests thereby preserving the function of HSCs (Lengefeld et al., 2021). This adds to our current understanding of the role of DNA damage and cellular dysfunction: In addition to DNA damage causing HSC dysfunction directly, DNA damage also drives HSC enlargement, which renders HSCs dysfunctional (Figure 2). Of note, DNA damage itself is not needed for the dysfunction of large HSCs, as increasing HSC size without DNA damage (e.g., mTOR overexpression) also drives the loss of function (Lengefeld et al., 2021). Overall, HSCs enlarge during aging after a series of transient cell cycle arrests, which ultimately causes their dysfunction.

Under physiological conditions, cells are able to return to their original size after size fluctuations by adjusting cellular growth and division speed (Cadart et al., 2018; Ginzberg et al., 2018; Bjorklund, 2019; Xie and Skotheim, 2020). Unexpectedly, like large HSCs (Lengefeld et al., 2021), yeast cells and primary human cells *in vitro* become larger with every division (Mortimer and Johnston, 1959; Mitsui and Schneider, 1976a; Mitsui and Schneider, 1976b; Bemiller and Miller, 1979; Yang et al., 2011; Lee et al., 2012) and do not shrink back to their original size. This is in line with the observation that many cell types enlarge with age *in vivo* and *vitro* (Roth et al., 1976; Treton and Courtois, 1981; Anversa et al., 1986; Melissari et al., 1991; Horsfall et al., 1994; Dyachenko etal., 2006; Martinelli et al., 2006a; Helms et al., 2010; Roh et al., 2013; Pucker et al., 2015; Mammoto et al., 2019; Delgado-González et al., 2020; Lengefeld et al., 2021; Yako et al., 2021). It is unclear why mechanisms of size homeostasis fail to reduce the size of these enlarging cell types.

In summary, an emerging picture in the field suggests a causal link between cell size and function in yeast, *in vitro* cell lines and *in vivo* systems. Furthermore, cellular enlargement aligns with the criteria of an aging hallmark. This highlights the importance of cell size for consideration in the design of aging studies.

## 3 Interplay between cellular enlargement of hematopoietic stem cells and other hallmarks of aging

Increased cell size was previously assumed to be a consequence of aging, rather than a causal factor (Johnson et al., 2013). Therefore, studies on aging may have overlooked cellular enlargement and did not evaluate a connection to other aging hallmarks. In this section, we discuss potential intersections between HSC enlargement and other hallmarks of aging, highlighting their overlaps and differences.

### 3.1 Cellular senescence

Senescent cells are irreversibly arrested in the cell cycle and are usually large in size (Biran et al., 2017). Large HSCs are always dysfunctional as they fail to build a blood system *in vivo* and display proliferative defects. This raises the question whether large HSCs are senescent and whether enlargement facilitates the senescent state (Lanz et al., 2022; Xie et al., 2022)? While little is known about this connection in HSCs, it has been shown that not all large HSCs are also senescent based on SA-β-gal production (Lengefeld et al., 2021). This indicates that HSC enlargement does not strictly induce cellular senescence. Nevertheless, based on the observation that senescent HSCs are always large, we speculate that large HSCs may be more prone to acquire the senescent program when exposed to additional damage. This is supported by the observation that larger human cells *in vitro* are as well more prone to senescence potentially *via* expansion of lysosomes and the endoplasmic reticulum (Cheng et al., 2021; Lanz et al., 2022). Overall, it will be important to further investigate how HSC enlargement increases the likelihood of entering the senescent state.

### 3.2 Telomere attrition and DNA damage

Telomere shortening and the accumulation of DNA damage during aging contribute to cellular dysfunction (Lopez-Otin et al., 2013)*.* Are telomere shortening and DNA damage also contributing to the dysfunction of large HSCs? As outlined above, DNA damage contributes to enlargement as it transiently halts HSC division to repair the DNA damage (Sperka et al., 2012). During this arrest, cell growth continues leading to HSC enlargement (Fingar et al., 2002; Neurohr et al., 2019). This enlargement drives dysfunction of HSCs. When growth is inhibited with rapamycin during this DNA damage, HSCs maintain their functionality (Lengefeld et al., 2021). Thus, if telomere shortening during DNA damage induced enlargement is crucial for their dysfunction, then we expect rapamycin to prevent telomere shortening and the associated accumulation of DNA damage. This is not the case. Rapamycin does not prevent DNA damage in HSCs and, so far, no protective effect of rapamycin on telomere length has been reported (Kawauchi et al., 2005; Qi et al., 2008; Ungar et al., 2011; Gopalakrishnan et al., 2018; Ferrara-Romeo et al., 2020). Additionally, other studies have indicated that it is still unclear whether telomere length plays a crucial role in HSC exhaustion (Allsopp et al., 2003): Telomeres become shorter during divisions of HSCs (Allsopp et al., 2001). However, at least in mice, HSCs become exhausted even when telomere shortening is prevented by telomerase overexpression (Allsopp et al., 2003) arguing that there are telomere-independent barriers to HSC function. Furthermore, we would like to speculate about the possible protective feature of enlargement. DNA-damage leads to enlargement and potentially permanent gene mutations and chromosomal aberrations. However, this enlargement in turn reduces the proliferation potential of these cells and thereby may prevent the propagation of potentially harmful features in the tissue that could facilitate, for example, a malignant transformation. In summary, DNA damage contributes to HSC dysfunction by enlarging their size, while telomere attrition seems to play a less dominant role than enlargement in causing HSC dysfunction.

### 3.3 Mitochondrial dysfunction

Mitochondrial volume typically scales with cell size in dividing cells supporting faithful cellular functions (Posakony et al., 1977; Rafelski et al., 2012; Miettinen and Bjorklund, 2016; Cheng et al., 2021; Lanz et al., 2021; Seel et al., 2022). For example, HSCs are able to scale mitochondria upon their activation (Ho et al., 2017). Changes in mitochondrial morphology and volume beyond this scaling are associated with cellular dysfunction (Chistiakov et al., 2014). Mitochondrial number and volume have often been quantified in old cells, but the results have been inconsistent. *In vitro*, some cell types display decreased mitochondrial volume (Stoll et al., 2011; Yako et al., 2021; Barilani et al., 2022), but most human cell lines increase their mitochondrial volume as they become larger and senescent (Lee et al., 2002; Passos et al., 2007; Koziel et al., 2011; Xie et al., 2011; Correia-Melo et al., 2016; Stab et al., 2016). It is noteworthy that increased mitochondrial volume does not necessarily translate into increased mitochondrial functions *in vitro* (Passos et al., 2007; Passos et al., 2010; Miettinen and Bjorklund, 2016). Furthermore, *in vivo* studies often reported decreased mitochondrial volume during aging (Markowska et al., 1994; Navarro and Boveris, 2004; Mathieu-Costello et al., 2005; Martinelli et al., 2006a; Addabbo et al., 2009; Leduc-Gaudet et al., 2015; Del Campo et al., 2018; Brown et al., 2021; Lengefeld et al., 2021). For example, in large HSCs mitochondrial volume decreases per unit volume (Lengefeld et al., 2021). Currently, the underlying reasons for these differences *in vitro* and *in vivo* are unclear. One possibility is that they are facilitated by different oxygen levels *in vivo* (hypoxic) and *in vitro* (hyperoxic). Nevertheless, these changes in mitochondrial volume during aging are associated with a decline in mitochondrial function, potentially driven by differences in fusion, fission and mitophagy (Mai et al., 2010; Korolchuk et al., 2017; Yako et al., 2021). Whether changes in mitochondrial morphology, volume and number during aging are the cause or consequence of cellular enlargement remains to be determined. In summary, while mitochondria volume and function scale with cellular size during faithful divisions, these parameters become uncoupled when cells enlarge during aging.

### 3.4 Loss of proteostasis

The scaling of protein levels with cell size is important for cellular function (Fraser and Nurse, 1978; Padovan-Merhar et al., 2015; Schmoller and Skotheim, 2015; Sun et al., 2020; Cheng et al., 2021; Lanz et al., 2021). Upon substantial enlargement of primary human cells *in vitro*, protein synthesis rates and ribosome levels decrease (Delarue et al., 2018; Neurohr et al., 2019). However, considering that disruptions of proteostasis contribute to HSC aging (Signer et al., 2014; Kruta et al., 2021), it was surprising that measurements of nucleolar size, cellular density, mTOR activity and protein synthesis in large HSCs indicated no loss of protein synthesis capacity (Lengefeld et al., 2021). While protein synthesis was unaffected by large HSC size, whether protein turn-over, folding state, aggregation and protein-protein interactions were impaired is unknown. Indeed, during aging, misfolded proteins contribute to the loss of cellular function (Lopez-Otin et al., 2013) and it will be important to analyze whether large stem cell size affects the accumulation of these misfolded proteins.

How enlargement of HSCs is related to the remaining hallmarks of aging like chromatin remodeling, genomic instability, nutrient sensing and intercellular communication is at this point unclear. It will be important to understand which aging hallmarks act in the same pathways as cellular enlargement to build a holistic picture of aging. Furthermore, initial experimental evidence suggests that enlargement can be reversed, which improved stem cell function (Lengefeld et al., 2021). This may open the possibility that other aging factors that are connected to cell size can be improved when shrinking stem cells.

## 4 Relation between cell size and aging-related functions: A fresh look at old studies

**FIGURE 3:**
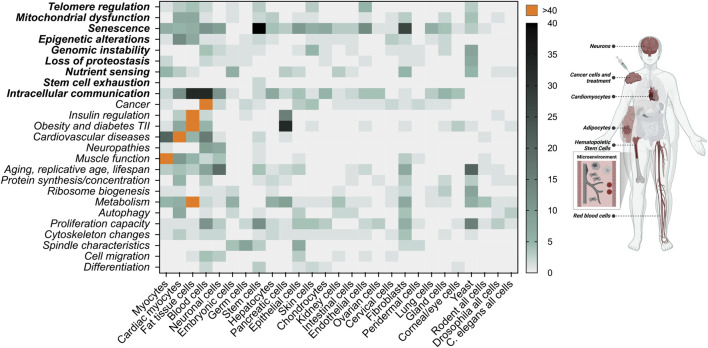
Literature research of connections between cell size and various cellular functions associated with aging. A total of 1,626 publications were manually collected using PubMed.gov and keywords related to cellular functions in combination with cell size in the abstract [“cell size” OR “cell volume” AND (category)]. These publications were categorized based on cell type and cellular function and presented as heat map. Aging factors are displayed in bold. AI methods for computation abstract search were considered, however this technology is currently limited for this purpose. We do not differentiate whether cellular size changes are associated with polyploidy, osmolarity or *in vivo/in vitro* studies. This figure is not a complete representation of the literature but gives the first unbiased insight that cellular size is connected to a large variety of cellular functions. On the left side of the image, we highlight the areas that we focus on in the main text. Created with BioRender.com.

As mentioned above, to evaluate whether cell enlargement qualifies as an aging factor/hallmark, it will be important to test for a causal relationship between enlargement and cellular dysfunction during aging in various different cell types. Interestingly, we can extract clues from numerous studies for how cell size potentially affects various cellular functions. Here, we present publications showing examples of cell size in association with different aspects of aging and pathologies (Figure 3). Whether size is causally associated with the corresponding changes is not addressed in these studies. Nevertheless, considering the recent findings that cellular enlargement manifests itself an aging factor, we take a fresh look at these studies in the next sections.

### 4.1 Enlargements as part of faithful cellular functions

Before we dive into the literature evaluating cellular size and age-related dysfunctions, we would like to point out examples of enlargement that are not associated with dysfunction.

*Cell cycle* - Cells usually double in size during the cell cycle to ensure that their daughter cells are the same size as the original mother cell after symmetric cell division (Lloyd, 2013). In muscle satellite cells, this enlargement can happen already prior to cell cycle entry and is called the G_alert_ state. This enables an accelerated entry into a proliferating state after repeated muscle injury (Rodgers et al., 2014).

*Differentiation* - Another example of enlargement as part of faithful cellular processes is differentiation, in particularly during development. For example, 1) keratinocyte enlargement during differentiation is proposed to enable the formation of an insoluble protein envelope at the plasma membrane (Watt and Green, 1981), 2) terminal differentiation drives enlargement of chondrocytes (hypertrophy) as part of skeletal tissue elongation (Cooper et al., 2013), 3) cardiomyocytes increase in size during heart development (Knaapen et al., 1996; Li et al., 1996), and 4) T cell activation requires profound changes in cell size (Kaesler et al., 2012). Furthermore, during cell manipulations that trigger differentiation, cells not only acquire a new cell identity but also a new cell size, which is optimal for this cell type. For example, overexpression of *RUNX3*, results in an increase in cell size and a block in faithful differentiation of erythrocytes (Menezes et al., 2022).

*Ploidy* - Changes in ploidy are generally associated with enlargement, but do not necessarily lead to dysfunction (Gillooly et al., 2015; Mu et al., 2020). For example, during adolescence, mouse acinar cells often become tetraploid and increase in size without losing their function (Xanthopoulos et al., 2008). On the other hand, polyploidization of hepatocytes occurs successively during postnatal development and again during advanced aging. The latter being associated with senescence, progressive loss of cell pluripotency and decreased replication capacity (Celton-Morizur and Desdouets, 2010; Wang et al., 2017).

*Apoptosis* - Cell volume decrease is characteristic of apoptotic cell death (Bortner and Cidlowski, 2002; Núñez et al., 2010). Cancer cells are able to prevent apoptotic shrinking by modulating the activity of ion channels (Lehen’kyi et al., 2011).

In conclusion, changes in cell volume are part of cellular processes and not necessarily associated with dysfunction. In the next part of this section, we will highlight findings, in which cellular enlargement is associated with aging, dysfunction and disease.

### 4.2 The mechanical microenvironment and cell size

Cell size is influenced by matrix stiffness, osmotic pressure and mechanical forces (Wang et al., 2020a). For example, cells substantially shrink when cultivated on substrates of increased stiffness (Guo et al., 2017; Xie et al., 2018; Yang et al., 2020). Interestingly, modulation of the external stiffness, and thereby cellular size, affects cellular function. For example, mesenchymal stem cells (MSCs) enlarge during aging *in vitro* and *in vivo*, which is accompanied by a senescent phenotype including high levels of p21, *β*-galactosidase, and SASP (Block et al., 2017; Yin et al., 2020; Liu et al., 2021). When MSCs are cultivated at an optimal substrate stiffness, they maintain their small size and proliferation capacity and do not become senescent (Kureel et al., 2019). Furthermore, cultivating large aged endothelial cells on substrate with increased stiffness rejuvenates these cells and reduces their size (Mammoto et al., 2019). The underlying mechanism is unknown. Together, this suggests that the environmental stiffness can modulate cellular size and thereby cellular functionality *in vitro*.

It is important to note that cells in 2D monocultures are exposed to a different microenvironment than in the *in vivo* 3D tissue context. *In vivo*, cells face the extracellular matrix (ECM), neighboring cells, biochemical and physical cues (Barthes et al., 2014), which all affect the environmental stiffness. As tissues have different solidity states, it is expected that stiffness varies between tissues and organs (Butcher et al., 2009). During aging *in vivo*, increased stiffness is associated with malignancy (Lieber et al., 2004; Butcher et al., 2009; Stearns-Reider et al., 2017; Segel et al., 2019; Ghosh et al., 2020), while stiffness decreases in some organs like the skin (Tissot et al., 2018; Runel et al., 2020; Lynch et al., 2022) and muscle tissues (Akagi et al., 2015; Yoshida et al., 2017; Alfuraih et al., 2019). We speculate that reduced stiffness contributes to cellular enlargement during aging. It will be of critical interest to analyze how microenvironmental forces affect cellular size and thereby cellular function during aging *in vivo*. In summary, the microenvironment modulates cell size and therefore likely cell function as well.

### 4.3 Cell size of red blood cells

Red blood cell distribution width (RDW) represents variability of the size distribution of red blood cells and has been used as a marker for multiple aging-related pathologies (Salvagno et al., 2015). RDW involves a change in mean corporal volume (MCV). An increase in MCV, meaning increase in red blood cell volume, correlates with aging (Hoffmann et al., 2015; Lippi et al., 2018) and numerous diseases like anemia, autoimmune diseases, cancer types, and cardio- and cerebrovascular diseases (Bessman et al., 1983; Li et al., 2017; Lippi et al., 2018; Katsaros et al., 2020). Interestingly, not only enlargement, but a deviation from optimal red blood cell size in general is associated with diseases, for example, various leukemia forms, Alzheimer’s disease, Parkinson’s disease, autoimmune disease, and macular degeneration (Pilling et al., 2017; Lippi et al., 2018; Katsaros et al., 2020; Kim et al., 2021).

It is unclear why red blood cells of many diseased individuals are large. The fact that red blood cells lack organelles, such as a nucleus and mitochondria, implies that the underlying mechanism driving dysfunction is different from the one in HSCs, in which these organelles are necessary for enlargement and dysfunction (Lengefeld et al., 2021). Furthermore, the correlation of RDW with aging-associated diseases has not been investigated for a causal relationship. Overall, RDW is a robust indicator of numerous diseases, but the origin of this relationship is insufficiently understood.

### 4.4 Cell size of adipocytes

Large size of adipocytes is associated with several pathophysiological conditions like reduced response to hormones, increased inflammation, impaired body metabolic regulation and insulin resistance, which is associated with type 2 diabetes (Bjorntorp et al., 1972; Bernstein et al., 1975; Weyer et al., 2000; Lundgren et al., 2007; Lonn et al., 2010; Yang et al., 2012; McLaughlin et al., 2014; Laforest et al., 2015; Stenkula and Erlanson-Albertsson, 2018; Liu et al., 2020). However, it is also suggested that it is the enlargement and not the absolute size that predicts insulin resistance (Johannsen et al., 2014). Interestingly, a recent model proposes that enlargement of adipocytes itself affects the interaction of the cell with the ECM. This activates integrin/Erk signaling and modulates gene expression (Farnier et al., 2002; Farnier et al., 2003). It will be exciting to see further studies evaluating the causality of cellular enlargement on adipocyte function.

Mean adipocyte size increases between middle and old age and then decreases with advanced age (Miller et al., 2017). Interestingly, this correlates with the risk for the onset of type 2 diabetes, which peaks at 45–65 years and lowers afterwards (Plummer et al., 2016; Sattar et al., 2019; Jacobs et al., 2020; Hu et al., 2021). Nevertheless, while smaller adipocyte size during advanced age correlates with a lower onset risk of type 2 diabetes, it coincides also with another unfavorable outcome: A result of decreased adipocyte size is the decline in fat depot size, which leads to the accumulation of fat outside adipose tissue like in the bone marrow, muscle and liver. This is associated with lipotoxic stress in the storage organs and lower organ function with age (Rosen and Bouxsein, 2006; Slawik and Vidal-Puig, 2006). Overall, enlargement of adipocytes predicts obesity-associated pathologies like type 2 diabetes and during aging, the shrinkage of adipocytes is likely associated with other pathologies.

### 4.5 The aging brain and Alzheimer’s disease

At old age, brain volume declines (Haug and Eggers, 1991; Resnick et al., 2003). Studies indicate that this is caused by a shrinking of neuronal cell size (Anderson et al., 1983; Terry et al., 1987; Diaz et al., 1999; Kuwahara et al., 2004; Martinelli et al., 2006b; Fernandez et al., 2007; Stark et al., 2007; Freeman et al., 2008; Verkhratsky et al., 2014; Popov et al., 2021). Nevertheless, in some areas of the brain neurons maintain size or enlarge with age (de Lacalle et al., 1991; Merrill et al., 2000; Martinelli et al., 2006b; Verkhratsky et al., 2014; Thulborn et al., 2016; Delgado-Gonzalez et al., 2020; Yako et al., 2021). Interestingly, during neurodegenerative diseases such as Alzheimer’s, neurons enlarge. Overexpression of the amyloid precursor protein causes enlargement of cortical neuron size (Oh et al., 2009) and larger neuronal cell lines are more Aβ-sensitive (Simakova and Arispe, 2007). This causes cellular toxicity and resembles cellular damage in Alzheimer’s diseased brains. Furthermore, an increase in cell size or neuronal hypertrophy has been reported in elderly that display no cognitive symptoms yet but markers of Alzheimer’s disease (Iacono et al., 2008). At later stages of Alzheimer’s disease progression, oligodendrocyte progenitor cells increase in size (Vanzulli et al., 2020) and neocortical neurons are enlarged in Alzheimer patients (Bundgaard et al., 2001; Pakkenberg et al., 2003). In summary, while most neuronal cell types shrink during aging, areas that contain neurons that enlarge might be especially prone to processes that result in symptoms of Alzheimer’s disease.

### 4.6 Cardiomyocyte hypertrophy

As the proliferative capacity of heart cells becomes severely restricted after birth, the postnatal increase of heart volume is achieved by cardiomyocyte hypertrophy (Li et al., 1996; Porrello et al., 2011). Heart hypertrophy coincides at the cellular level with nuclear polyploidization, multinucleation, and cellular growth (Derks and Bergmann, 2020). While cardiomyocyte hypertrophy is associated with development and adjustment to exercise (Ellison et al., 2012), it also predicts numerous cardiovascular diseases like ischemic disease, hypertension, heart failure, and valvular disease (Wang et al., 2013; Akhondzadeh et al., 2020; Peter et al., 2016). This raises an important question: What is the difference between physiological and pathological cellular hypertrophy?

Cellular growth driven by the IGF1/PI3K/Akt1 pathways facilitates heart enlargement after exercise, while signaling *via* G_q_ protein-coupled receptors, reduced blood supply, oxidative stress, inflammatory processes and Ca^2+^ abnormalities drive cellular growth and enlargement of the heart associated with pathology (Weeks and McMullen, 2011; Oldfield et al., 2020). Thus, in the case of the heart, enlargement is associated with opposite outcomes and thus, it seems unlikely that they are driven by enlargement itself.

Nevertheless, there are some interesting differences to be noted: For example, enlargement after exercise changes the cellular shape in a different way than during disease (Kuo et al., 2012; Peter et al., 2016). Furthermore, it could be that pathological hypertrophy enlarges cardiomyocytes beyond a size-threshold due to extensive growth. This is supported by the observation that cardiomyocytes after exercise are smaller than cardiomyocytes under diabetic stress (Stolen et al., 2009). While many cardiomyocytes become polyploid during pathological enlargement, whether this enlargement is always accompanied with an increase in DNA copy number is unclear. Interestingly, in yeast, enlargement without increasing DNA copy number results in cytoplasmic dilution and dysfunction (Neurohr et al., 2019). Thus, it will be interesting to investigate whether mechanisms like cytoplasmic dilution contribute to the dysfunction of cardiomyocytes upon pathological enlargement and whether polyploidization is a compensating mechanism to prevent this functional loss. Indeed, polyploidy has been hypothesized as a mechanism to increase metabolic and growth capacity of cells (Frawley and Orr-Weaver, 2015; Mu et al., 2020). Furthermore, if it is true that pathological enlargement facilitates changes in ploidy, this could also include changes like large genomic aberrations and aneuploidy. These genomic defects may also facilitate unwanted downstream effects. Indeed, aneuploidy is a distinct feature of cancer cells (Ben-David and Amon, 2020). Overall, being of the right size is important for cardiomyocytes (Ren and Brown-Borg, 2002) and hypertrophy is a crucial characteristic of adjusting to exercise and pathology.

### 4.7 Cell volume and cancer

Heterogeneity of cell size and shape are often observed in tumors and referred to as pleomorphism (Manocha et al., 2003; Terzakis et al., 2005). This raises the question of whether a failure of size regulation is facilitating cancer progression. Some research suggests that size heterogeneity of cancer cells provides the highest malignant potential (Kawada et al., 1994; Manukyan et al., 2021). Furthermore, the population of small cells within tumors raise attention. Small prostate cancer cells build more malignant tumors than large ones (Li et al., 2015). Mutated *STAT3* is a crucial cancer facilitator and leukemia patients with *STAT3* mutations carry smaller blood cells than patients without these mutations (Tanahashi et al., 2016) suggesting that the smaller cells might facilitate cancer progression. It is also speculated that cancer stem cells are small, just like most stem cells (Berardi et al., 1995; Colter et al., 2001; Young et al., 2004; Virant-Klun and Stimpfel, 2016). The small size could result from the fast proliferation of cancer cells, leaving little time for growth during these short cell cycle durations (Lloyd, 2013; Wright and Schneider, 2014). On the other hand, a small size might support the cancerous potential of the cells. One possibility is that size affects the migration behavior of cancer cells (Leal-Egana et al., 2017; Wang et al., 2020b). Overall, the literature provides interesting clues that a deviation from the optimal cell size is associated with cancer, but whether and how size modulates malignancy is unclear.

Solid tumors are usually an assembly of distinct cell types including infiltrating immune cells, each owning their own cell size (Ginzberg et al., 2015). Thus, the size heterogeneity found in cancer tissues could reflect the tumor’s composition of various cell types (Shembrey et al., 2019) and not a failure in size regulation of the cells that originated the cancer. However, cell size could still serve as a diagnostic marker for certain types of cancers. For example, for acute lymphoblastic leukemia, the significance of cell size as a prognostic indicator is heavily investigated (Mathe et al., 1971; Pantazopoulos and Sinks, 1974; Murphy et al., 1975; Oster et al., 1976; Dubner et al., 1978; Ghani and Krause, 1986; Olcay et al., 1999). Further research is needed to determine whether cell size is a reliable predictor of malignancy. Investigating the potential link between cellular size and cancer potential will be crucial for the development of new therapies for cancer treatments.

### 4.8 Effect of cell cycle inhibitors on cell size during treatment approaches

As discussed, an arrest in cell cycle causes cellular enlargement (Fingar et al., 2002; Neurohr et al., 2019; Lengefeld et al., 2021). Cell cycle inhibitors like palbociclib, dinaciclib, seliciclib, ribociclib, and abemaciclib therefore enlarge cell size. These kinds of drugs are already successfully used in clinical trials or are even FDA-approved for cancer treatment. As enlargement can cause dysfunction, this could contribute to the inhibitory success of these cell cycle inhibitors in cancer treatments. Recent studies provide important insights into this topic. Uncoupling cell growth and proliferation in cancer cells by arresting them in G_1_ phase leads to toxic overgrowth and senescence (Wilson et al., 2021; Crozier et al., 2022; Foy et al., 2022; Manohar et al., 2022). Furthermore, keeping patient-derived glioma stem cells small (rapamycin treatment) during cell cycle arrest (palbociclib) prevents their entry into a senescence state (Morris-Hanon et al., 2019). Thus, growth and enlargement, in addition to cell cycle arrest, effectively inhibit cancer cells from proliferating and cancer treatments using cell cycle inhibitors may therefore not be combined with rapamycin.

Cell cycle inhibitors like palbociclib have also been investigated as treatment for other pathologies: mast cell-mediated allergy (Hou Y. B. et al., 2019), diabetic cardiomyopathy (Wang et al., 2019), pulmonary arterial hypertension (Weiss et al., 2019), replication of herpes virus (Badia et al., 2016), systemic sclerosis (Yamamoto et al., 2022) and spinal muscular atrophy (Hor et al., 2018). It will be important to evaluate how arrest-induced growth and enlargement affect the outcome of these diseases. Overall, this highlights the importance of understanding how growth and enlargement contribute to the multilayered effects of treatment drugs.

#### 4.9 Cell size and lifespan

Cell enlargement is associated with cellular aging. Does cell size also influence organismal lifespan? A recent study found a reliable predictor of lifespan across 24 mammalian species: Pancreatic acinar cell volume inversely correlates with lifespan of different species (Anzi et al., 2018). Noting that ploidy was not accounted for in this correlation, the study suggests that these results might apply to multiple other tissues. Mice with a reduced body size and hereditary dwarfism are longer-lived than larger mice (Roberts, 1961; Eklund and Bradford, 1977; Brown-Borg et al., 1996; Miller et al., 2000; Flurkey et al., 2002; Miller et al., 2002). Interestingly, cardiomyocytes from old dwarf mice are smaller compared to their wild-type counterparts (Helms et al., 2010). While these smaller cardiomyocytes are associated with cardiac contractile defects (Ren and Brown-Borg, 2002), they also display reduced extracellular collagen, which is protective against heart disease (Helms et al., 2010) and may have improved cardiac function during aging (Li et al., 2007; Reddy et al., 2007). It remains to be determined whether small cardiomyocyte size is contributing to longevity. We note that there are cell types that are exceptions: for example, human neurons and astrocytes are larger than the ones in mice (Herculano-Houzel et al., 2006; Oberheim et al., 2009; Rostock et al., 2018), yet humans live longer than mice. Overall, cellular size could serve as an indicator of lifespan when choosing the correct cell type.

## 5 Summary and outlook

Researchers unraveling the process of aging and its associated diseases have revealed that aging is associated with great phenotypic variation, which is driven by several aging hallmarks. Now, it crystalizes that one of these hallmarks is cellular enlargement, at least for HSCs. Recent data suggest that cellular enlargement is not only correlated with aging, but also causally contributes to the functional decline of HSCs during aging. While cells have an innate ability to regulate their cell size, upon age-dependent enlargement this regulatory mechanism fails. This new concept of cellular enlargement driving dysfunction during aging has been tested for HSCs. Interestingly, numerous other cell types have also been observed to enlarge during aging (Hayflick and Moorhead, 1961; Mitsui and Schneider, 1976a; Mitsui and Schneider, 1976b; Treton and Courtois, 1981; Martinelli et al., 2006a; Mammoto et al., 2019). This raises the possibility that cellular enlargement contributes to aging in other cell types. Moving forward, the research community will benefit from further experiments providing critical evidence of whether cellular enlargement is cause or consequence of aging in other stem cell types and differentiating cells. Furthermore, 1) researchers working with rare clinical samples should consider whether a simple measurement of cellular size will lead to important insights on aging, 2) categorizing cell types by cell size might explain ageing-related heterogeneity of samples and even reveal new pathways, 3) cancer treatments might cause the desired effect or undesired side-effects by changing cell size, and 4) enlargement of certain cell types might explain aging-associated cellular dysfunctions. As a result, cellular enlargement might affect several processes related to physiology, aging and disease that have so far not been connected to cell size. Understanding how cellular enlargement relates to aging and disease will help to explain previous observations, direct research, and support the development of new therapies for aging-related diseases.

Changes in the cell’s volume during various processes are necessary for the cell to function, for example during the cell cycle, development, and differentiation. Interestingly, the literature also provides an overwhelming number of publications reporting a correlation between cell size and various cellular dysfunctions. Our literature analysis revealed areas, which are connected to cell size like cancer, cardiovascular diseases, obesity, autoimmune diseases and Alzheimer’s disease. Other factors, like the ECM stiffness, caught our attention for having a potential effect on cell size *in vivo*. Interestingly, not only enlargement of cells is associated with dysfunctions, but a general deviation from the optimal cell size affects cancer progression, diseases associated with red blood cells and potentially neurodegenerative aging. Furthermore, cell size might be a predictor of lifespan, cancer, type 2 diabetes, heart failure and Alzheimer’s disease. A systematic documentation of cell size of different cell types during health, disease and aging may allow to define a threshold beyond which cells are more likely dysfunctional. Overall, this could indicate that there is a relationship between cell size and cellular failure in different cell types of multicellular systems. However, this hypothesis needs testing. Now, we encourage researchers to test a potential causal connection between cell size, pathology and aging, to analyze the value of cell size as a prognostic marker for diseases, and to evaluate whether cellular enlargement qualifies as a new aging hallmark. These new insights will affect research areas that have so far not considered the importance of cell size.
